# *Experimental Physiology* special issue: ‘Mechanotransduction, muscle spindles and proprioception’

**DOI:** 10.1113/EP091431

**Published:** 2023-12-31

**Authors:** Stephan Kröger

**Affiliations:** ^1^ Department of Physiological Genomics, Biomedical Center Ludwig‐Maximilians‐Universität Planegg‐Martinsried Germany

This special issue of *Experimental Physiology* contains a collection of 13 manuscripts based on oral presentations presented at the meeting on ‘Mechanotransduction, Muscle Spindles and Proprioception’, which took place at the Ludwig‐Maximilians‐Universität in Munich in July 2022. The participants included >30 speakers from Taiwan, Canada, Australia, the USA, Israel and from all over Europe and the UK. In this Editorial, I want to present a brief personal view of how the idea to organize this meeting emerged and put the individual publications of this issue into perspective.

Proprioception is essential for all coordinated movements and required for perceiving the position of the body in space (Proske & Gandevia, 2012). Given that Aristotle (384–322 BC) first defined smell, sight, touch, taste and hearing as the five senses, proprioception has often been coined the ‘sixth’ sense, despite >20 senses being known, with the exact number of senses remaining elusive. Although proprioception is an integrative system that processes information from a combination of peripheral sensory input, including muscle length and tension, joint angle and skin stretch (Macefield & Knellwolf, 2018), the key components of this intricate system are muscle spindles (Matthews, 2015). Embedded in almost every skeletal muscle, these primary proprioceptive sensory organs relay constant information about muscle tone and length to the CNS (Kröger, 2018; Proske & Gandevia, 2012). From this information, the CNS processes the spatial position and motion of the body in space, a process crucial for motor control, voluntary movement, posture and a stable gait (Kröger, 2018; Kröger & Watkins, 2021).

During its prime time in the 1950s and 1960s, muscle spindles were at the forefront of sensory physiology, and many basic principles were discovered using muscle spindles, including, for example, the rate coding of stimulus intensity (Adrian & Zotterman, 1926). For sensory physiology, muscle spindles were similar to what the neuromuscular junction had been for the discovery of the basic principle of synaptic transmission. In fact, many scientists working on the neuromuscular junction also worked on muscle spindles, including Sir Charles Sherrington, Steven Kuffler and Sir Bernhard Katz (see e.g., Hunt & Kuffler, 1951a, 1951b; Katz, 1950; Sherrington, 1907). The tenuissimus muscle from the cat had become a classic preparation, and muscle spindle function is probably still best characterized in this particular muscle (for more historical information regarding muscles as sense organs required for muscle tone and posture, see Molnar & Brown, 2010). However, in the 1970s and 1980s, the interest in muscle spindles declined, and the muscle spindle/proprioception field mostly bypassed the beginning of the ‘molecular age’. This has changed in the past decade, leading to the identification of new, often surprising functions, which have extended the essential role of muscle spindles far beyond sensing the position of the body in space (Kröger, 2018). These rather recent discoveries include, for example, the identification of the Piezo2 channel as the main mechanotransduction channel in muscle spindles (Woo et al., 2015), the discovery that in several diseases muscle spindle function is directly or indirectly affected (Kröger & Watkins, 2021), the strong influence of muscle spindles on the skeletal system (Blecher et al., 2018), the identification of proprioceptor subtypes (Dietrich et al., 2022), the role of muscle spindles in maintaining locomotor robustness (i.e., the ability to cope with perturbations; Santuz & Akay, 2023) or the elucidation of the molecular basis of the differentiation signals during muscle spindle and sensory neuron development (Cheret et al., 2013; Hippenmeyer et al., 2002). Monoclonal antibodies in combination with confocal microscopy have replaced the silver impregnation techniques and are now widely applied to characterize muscle spindles on the molecular level. Single‐cell or single‐nucleus sequencing of intrafusal fibres and of the sensory and the motor neurons or the proteomics analysis of muscle spindles provide essential platforms for the design of specific molecular markers for particular cell populations (Blum et al., 2021; Bornstein, Heinemann‐Yerushalmi et al., 2023; Dietrich et al., 2022; Kim et al., 2020; Oliver et al., 2021).

Unfortunately, many of the scientists who rediscovered and revitalized the muscle spindle field and proprioception during the last decade came from different areas of neuroscience and did not know each other. Therefore, the exchange of ideas and (equally important) the passing on of the information to young scientists was difficult. There was simply no meeting where the new findings and their functional implications could be discussed.

After visiting Guy Bewick and Bob Banks in Aberdeen and Katherine Wilkinson in San José, we agreed that a meeting should be held where classic muscle spindle knowledge would merge with the latest new concepts, with the overall scientific goal being to provide a forum that united international experts in the field of mechanotransduction, muscle spindle function and higher‐order processing of proprioceptive information to share knowledge, exchange ideas and develop new effective experimental strategies by initiating collaborations between researchers with different areas of expertise.

With this focus in mind, leading scientists were invited, and almost everybody agreed to come to Munich, further demonstrating the need for a meeting with this focus. The meeting itself started with a fantastic introduction into the field from a historical perspective by Bob Banks. He summarized 50 years of research on the structure and function of the mammalian muscle spindle, its intrafusal muscle fibres and their sensory and motor innervation. In addition, he presented new quantitative morphological results on the equatorial nuclei of intrafusal muscle fibres and of the primary sensory endings in relationship to passive stretch of the spindle (Banks, 2024).

The first session was on mechanotransduction (i.e., the conversion of a mechanical stimulus into a change of the membrane potential). The Piezo2 channel is the primary mechanosensitive channel in muscle spindle mechanotransduction, and loss‐of‐function mutations in humans result in muscular atrophy, with perinatal respiratory distress, arthrogryposis and scoliosis (Chesler et al., 2016; Delle Vedove et al., 2016). However, other channels must also be considered in the initial mechanotransduction event, including the different acid‐sensing ion channel (ASIC) subtypes. Accordingly, several speakers covered the role of the ASICs in muscle spindle function. For example, using substrate deformation‐driven neurite stretch and micropipette‐guided ultrasound in knockout mouse models of ASIC subtypes, researchers in Chih‐Cheng Chen's laboratory analysed the roles of ASIC3 and ASIC1a in muscle spindles and other organs relying on mechanotransduction (Lin et al., 2024). Using in vivo proprioception‐related behavioural assays and ex vivo electrophysiological analyses of muscle spindles, mice lacking ASIC2 were shown to display impairments in muscle spindle responses to stretch and motor coordination tasks, demonstrating that ASIC2s are required for normal muscle spindle function (Bornstein, Watkins et al., 2024). Moreover, these mice also revealed skeletal deficits, including an impaired spinal alignment (Bornstein, Watkins et al., 2024), further supporting the important role of proprioception for the development or maintenance of the skeletal system. Collectively, these studies identify ASICs as key components in mechanosensation.

Given that the Piezo2 channel is rapidly adapting, but the response of muscle spindles to stretch is slowly adapting, there is a requirement for additional molecular elements to maintain firing during stretch. One such additional element could be glutamate. In fact, spindle afferent sensory endings contain glutamate‐filled synaptic‐like vesicles that are released in a stretch‐ and calcium‐dependent manner (Bewick, 2015). Consistently, blocking glutamate packaging into vGluT1‐containing vesicles or transgenic knockout of one allele of the vesicular glutamate transporter vGluT1 decreases muscle spindle afferent static but not dynamic sensitivity (Than et al., 2021). This has led to a model of mechanotransduction in which calcium entering through the Piezo2 channel can cause the release of glutamate from synaptic‐like vesicles, which then helps to maintain afferent depolarization and firing. In his presentation, Guy Bewick gave evidence that the glutamate‐sensitive channel contributing to the sustained firing in muscle spindles, initially identified as a metabotropic glutamate receptor coupled to phospholipase D, is the homomeric metabotropic GluK2 (Thompson et al., 2024). Pharmacological, immunohistochemical and biochemical evidence and its expression in primary proprioceptive sensory terminals are consistent with this hypothesis.

Muscle spindle function is impaired in many pathological situations (Kröger & Watkins, 2021). A rare autosomal recessive disease causing a selective loss of specific sensory neurons, leading to greatly elevated pain and temperature thresholds, poor proprioception, marked ataxia and disturbances in blood pressure control is hereditary sensory and autonomic neuropathy type III (HSAN III), also known as familial dysautonomia or Riley–Day syndrome. Owing to the absence of functional muscle spindles, stretch reflexes are absent throughout the body, leading, among other things, to a greatly compromised proprioception at the knee joint, loss of proprioceptive acuity at the knee and a severe gait impairment. Surprisingly, proprioception is normal at the elbow, but the patients perform poorly in the finger‐to‐nose test and in the Purdue pegboard task, compared with age‐matched healthy control subjects (Macefield et al., 2024). The most likely explanation for the differential effect of the mutation is that, in contrast to patients with a large‐fibre neuropathy (Cole & Sedgwick, 1992) in which cutaneous and muscle afferents are lost, HSAN III patients have access to sensory information from cutaneous mechanoreceptors in the skin. In the future, diseases like this might allow the more accurate analysis and treatment of the symptoms after selective loss of the sensory feedback provided by muscle spindles.

Classical proprioceptors (muscle spindles and Golgi tendon organs) are mostly absent in human facial muscles (Cobo et al., 2017; Omstead et al., 2024) and in the extraocular muscles of most mammals. In contrast, palisade endings are present in some mammalian extraocular muscles, and it was suggested that they are sensory structures, which might substitute functionally for the absence of muscle spindles. This would explain experimental studies indicating that the brain has access to information on eye position. Likewise, palisade endings exhibit structural characteristics in common with the classical proprioceptors. Interestingly, the palisade endings also contain many clear vesicles and the molecular machinery for the calcium‐mediated exocytosis of acetylcholine (Blumer et al., 2020). These results suggest that palisade endings are more complex, that is, that they might not only provide sensory information from the eye to the brain but might also have motor features (Blumer et al., 2024).

Two presentations considered the role of muscle spindles in the perception of musculoskeletal pain. Although muscle spindles are non‐nociceptive, their sensory neurons express several ASICs, which are dual‐function proteins for proton sensing and mechanosensing. Thus, it was hypothesized that these channels might have a role in the development of pain associated with tissue acidosis (Lee & Chen, 2024). Consistent with this hypothesis, clinical evidence has shown a proprioceptive deficit among patients with chronic pain or fibromyalgia and it has been found that training in proprioception is beneficial for pain relief (Lee & Chen, 2024). One possibility is that proprioception is affected by microdamage to muscle spindles or by acidosis caused, for example, by eccentric and concentric exercise, respectively (Lee & Chen, 2024; Lund et al., 2010; Sas et al., 2024). Large‐diameter primary afferents, such as those innervating muscle spindles, become hyperexcitable and develop spontaneous ectopic firing (i.e., at places away from the sensory ending within the muscle spindle capsule) in conditions leading to neuropathic pain. Astrocytes, which are known to be activated in pain conditions, might contribute to the generation of ectopic firing, opening the possibility that cross‐talk between proprioceptive and nociceptive pathways might occur in the periphery, within the spindle capsule (Sas et al., 2024). Clearly, despite limited evidence supporting a direct role for proprioceptors in nociception, the effect of acid signalling, ASIC signalling, structural damage of muscle spindles and astrocyte–sensory neuron cross‐talk on proprioception deserve further investigation, because understanding the role of proprioceptors in pain might provide new approaches for the development of effective treatments for chronic musculoskeletal pain.

In his presentation, Huub Maas addressed the question of whether muscle spindle activity attributable to stretch is restricted to the homonymous muscle or whether adjacent joints/muscles are also affected (Maas & Noort, 2024). This study concluded that changing the length of one muscle affects the firing behaviour of muscle spindles in a neighbouring muscle. Moreover, dynamic knee joint rotations of 15° caused changes in the firing rate of proprioceptive afferents of the soleus muscle. Thus, muscle spindles provide the CNS with information not only about the change in length of the homonymous muscle but also about the condition of adjacent joints that the muscle does not span (Maas & Noort, 2024).

Given that muscle spindles are part of a complex biological system and that their function is affected by the biophysical properties of the muscle, analysis of these biophysical properties is vital to our understanding of muscle spindle function in vivo, in particular as these properties change with age. For example, there is evidence that muscle fascicle biomechanics are more compliant in older adults, leading to decreased proprioceptive ability that affects movement and posture. Accordingly, researchers in Lena Ting's laboratory investigated the effects of increased tendon compliance on muscle spindle feedback during movement. Her results suggest that tendon compliance might not attenuate rapid bursts from muscle spindles at the onset of stretch but might decrease firing responses during movement, and these changes are not explained fully by changes in muscle fascicle length (Abbott et al., 2024).

Computational models are crucial to test functional hypotheses and to predict behaviour. For instance, this is required if one wants to provide amputees with physiologically relevant proprioceptive feedback from their prosthesis. Accordingly, several presentations centred on computational biophysical models of muscle spindle function. Researchers in Tim Cope's laboratory developed a computational model of the mammalian muscle spindle that, for the first time, integrates the asymmetric distribution of known voltage‐gated ion channels with neuronal architecture to generate realistic firing profiles to determine how these parameters influence mechanosensory encoding by muscle spindle afferents (Housley, Powers et al., 2024). Using this model, they showed that neuronal architecture and the asymmetric subcellular distribution and ratios of voltage‐gated ion channels are a complementary and, in some instances, orthogonal means to regulate muscle spindle encoding of stretch stimuli.

Another study used a biophysical model to ask how muscle cross‐bridge dynamics shape the information that can be encoded by intrafusal muscle fibres within the muscle spindle (Simha & Ting, 2024). Results from this study show that both actin and myosin dynamics and their interactions can shape muscle spindle sensory signals. Moreover, the incorporation of the actin and myosin dynamics into the computational model is necessary for the history‐dependent muscle spindle firing properties to be more in line with experimental observations. The tuned muscle spindle model shows that non‐linear and history‐dependent muscle spindle firing properties in response to sinusoidal stretch protocols emerge from intrafusal cross‐bridge dynamics.

In summary, the presentations covered many and diverse aspects of proprioception and muscle spindle function. This broad spectrum clearly contributed to the great success of the meeting, and the enthusiasm about the success was articulated by many individual comments of the participants during and after the meeting and is also reflected by the ability to publish many of the presentations in this issue of *Experimental Physiology*. The talks were truly excellent, and the discussions were friendly but directly to the point and sometimes controversial (Housley, Gardolinski et al., 2024). In addition, a number of collaborations were established during the meeting. Thus, the main objectives of the meeting to bring scientists from many different areas of proprioception together, discuss their findings and initiate synergies were achieved. Another meeting with a similar agenda is therefore planned in Munich in July 2024.

It only remains to thank all those involved in helping me to initiate and organize the meeting. Most importantly, I would like to thank colleagues in my laboratory at the Biomedical Center of the LMU Munich (Bridgette Watkins, Corinna Haupt, Zoi Gioga, Arlind Lamcaj and Jürgen Schultheiss), without whose hard work, dedication and co‐ordination activities the conference simply would not have happened. I would also like to acknowledge thankfully the financial support from the German Research Foundation (DFG) and from our industrial sponsors. Finally, I would like to thank Bob Banks, Guy Bewick and Katherine Wilkinson together with the team from *Experimental Physiology*, especially Diana Jones and Alex Steward, for their unstinting support and patience during the compilation of these papers.

## AUTHOR CONTRIBUTIONS

Stephan Kröger wrote this Editorial. He is accountable for all aspects of the work including questions related to the accuracy or integrity of any part of the work. All persons designated as authors qualify for authorship, and all those who qualify for authorship are listed.

## CONFLICT OF INTEREST

The author declares that he has no known competing financial interests or personal relationships that could have appeared to influence the work reported in this paper.

## FUNDING INFORMATION

None.
